# Gait disorders in the elderly and dual task gait analysis: a new approach for identifying motor phenotypes

**DOI:** 10.1186/s12984-017-0218-1

**Published:** 2017-01-31

**Authors:** Bernard Auvinet, Claude Touzard, François Montestruc, Arnaud Delafond, Vincent Goeb

**Affiliations:** 1 Rheumalogy Unit, Polyclinique du Maine, 4 Avenue des Français Libres, F 53010 Laval, France; 2 Geontology Unit, Centre Hospitalier de LAVAL, Rue du haut rocher, F 53000 Laval, France; 3 eXYSTAT SAS, 34 rue Victor Hugo, F 92240 Malakoff, France; 4 Radiology Unit, Polyclinique du Maine, 4 Avenue des Français Libres, F 53000 Laval, France; 5 Rheumatology Department, University Hospital, F 80054 Amiens, France

**Keywords:** Gait disorders, Elderly, Gait analysis, Dual task paradigm, Motor phenotypes

## Abstract

**Background:**

Gait disorders and gait analysis under single and dual-task conditions are topics of great interest, but very few studies have looked for the relevance of gait analysis under dual-task conditions in elderly people on the basis of a clinical approach.

**Methods:**

An observational study including 103 patients (mean age 76.3 ± 7.2, women 56%) suffering from gait disorders or memory impairment was conducted. Gait analysis under dual-task conditions was carried out for all patients. Brain MRI was performed in the absence of contra-indications. Three main gait variables were measured: walking speed, stride frequency, and stride regularity. For each gait variable, the dual task cost was computed and a quartile analysis was obtained. Nonparametric tests were used for all the comparisons (Wilcoxon, Kruskal-Wallis, Fisher or Chi^2^ tests).

**Results:**

Four clinical subgroups were identified: gait instability (45%), recurrent falls (29%), memory impairment (18%), and cautious gait (8%). The biomechanical severity of these subgroups was ordered according to walking speed and stride regularity under both conditions, from least to most serious as follows: memory impairment, gait instability, recurrent falls, cautious gait (*p* < 0.01 for walking speed, *p* = 0.05 for stride regularity). According to the established diagnoses of gait disorders, 5 main pathological subgroups were identified (musculoskeletal diseases (*n* = 11), vestibular diseases (*n* = 6), mild cognitive impairment (*n* = 24), central nervous system pathologies, (*n* = 51), and without diagnosis (*n* = 8)). The dual task cost for walking speed, stride frequency and stride regularity were different among these subgroups (*p* < 0.01). The subgroups mild cognitive impairment and central nervous system pathologies both showed together a higher dual task cost for each variable compared to the other subgroups combined (*p* = 0.01). The quartile analysis of dual task cost for stride frequency and stride regularity allowed the identification of 3 motor phenotypes (*p* < 0.01), without any difference for white matter hyperintensities, but with an increased Scheltens score from the first to the third motor phenotype (*p* = 0.05).

**Conclusions:**

Gait analysis under dual-task conditions in elderly people suffering from gait disorders or memory impairment is of great value in assessing the severity of gait disorders, differentiating between peripheral pathologies and central nervous system pathologies, and identifying motor phenotypes. Correlations between motor phenotypes and brain imaging require further studies.

**Electronic supplementary material:**

The online version of this article (doi:10.1186/s12984-017-0218-1) contains supplementary material, which is available to authorized users.

## Background

Gait disturbances and cognitive impairment in elderly people are two major issues that widen the gap between overall life expectancy and disability-free life expectancy [1]. Gait abnormalities are one of the main causes of chronic disability in the elderly population, and their incidence varies greatly with aging, since they occur in approximately 35% of adults aged over 70 years [2] and in 72% of people aged over 80 years [3]. Gait abnormalities can lead to mobility limitations, which are associated with loss of autonomy, reduced quality of life, increased fall risk, repeated hospitalizations, and premature death [4]. Moreover, gait performance is also a predictor for survival [5], cognitive decline [6], fall status [7], and quality of life [8]. Since the landmark article of Sudarsky [9], who was one of the first to consider gait disorders in the elderly as a topic worthy of investigation, few clinical studies have examined the burden of gait disorders in the elderly from a clinical approach. Gait disorders are often referred to as gait instability in the relevant literature. However, in clinical practice, gait disorders include a large number of situations, in which gait instability may be expressed by an unsteady gait without falls, memory complaints, or obvious gait disorders.

Cognitive decline is another cause of chronic disability in elderly people and well known to be an independent risk factor for falls, disability, and dementia. Its prevalence increases with age: 25% of adults over 65 years have cognitive impairments [10]. The co-occurrence of gait disturbances and cognitive impairment may exist due to a common underlying pathology [11–13]. Moreover, it has long been known [14] that there is a direct relationship between cognitive impairment severity and increased gait abnormalities, in both elderly people [15] and younger adults [16]. The control of walking depends on shared brain networks dedicated to cognition (attention, executive functions, working memory) and motor control [1, 17].

To assess the interactions between gait and cognition, the dual task paradigm [1, 7, 18] has become the reference method. During the dual task test the subject performs an attention-demanding task, whilst walking at a self-selected speed. Quantitative Gait assessments are mainly provided by instrumented walkway [6, 19], or accelerometric recordings [20–22]. Quantitative analysis of gait can be reduced to three principal domains which include pace, rhythm, and variability [6]. However, raw gait variables including walking speed, stride frequency, and stride regularity can also be used in this analysis [20, 21].

The decrease in gait variables walking speed, stride frequency, and stride regularity under dual task enables the calculation of the dual task cost (DTC). These gait modifications are interpreted as the increased cost of involvement of cortical attention processes whilst walking. Such DTC may reveal subtle brain impairments [23], and may be related to attention and executive function efficiency [24]. Therefore, gait assessment under dual-task conditions has been proposed in clinical settings as a window into brain function in the early stages of cognitive decline [1]. One may conclude that gait analysis under single task (ST), provides firstly a measure of the motor status (or motor function). The DTC measured under the dual task (DT) method provides a measure of the cognitive resources [18, 25] dedicated to the control of gait. Given that a high DTC is linked to cognitive involvement, we hypothesized that DTC measurement would allow the differentiation between gait disorders of central nervous system origin from those due to peripheral diseases. Lastly, based on the evolution of gait variables under dual-task conditions, specific motor phenotypes were described and found to be related to central nervous system pathologies [26], or to brain MRI abnormalities [27, 28].

The aim of this observational prospective clinical study was: 1. To assess the value of gait instability as a clinical symptom, 2. To quantify gait disorders by means of the DTC in order to differentiate between peripheral pathologies and central nervous system (CNS) pathologies, 3. To identify motor phenotypes according to the dual task cost for stride frequency and gait regularity (authors hypothesize that stride frequency and stride regularity can evolve independently in older patients based on unpublished data), 4. To search for correlations between these motor phenotypes and conventional brain MRI findings.

## Methods

### Trial design

This study is a prospective observational study that investigates the clinical value of gait instability symptoms in the elderly patients and quantifies gait disorders under ST and DT. In order to highlight the clinical impact of gait disorders as well as the contribution of cognitive decline to gait disorders, and to provide a better understanding of the underlying pathologies, outpatient consultations dedicated to gait disorders in elderly patients were launched simultaneously in the rheumatology unit of a private hospital and the gerontology department of a public hospital. During these outpatient consultations, an ambulatory gait analysis was carried out under single and dual-task conditions, selecting 3 major gait variables: walking speed, stride frequency and stride regularity. Due to the broad diversity of pathology that may be involved in gait disorders, outpatient consultations were linked to a radiology unit and to a number of specialists, who might intervene in elderly patients with gait disorders, such as neurologists, neuropsychologists, otolaryngologists, cardiologists, psychiatrists, etc.; hence the Gait Instability Network (GIN) was formed. Scientific meetings with General Practitioners were held to highlight the strong relationship between gait and cognition in the elderly and to inform them of the different services provided by these outpatient consultations. The study was approved by the Local Ethical Committee of Nantes University, and procedures were carried out in accordance with the standards listed in the Declaration of Helsinki, 1964.

### Participants

Patients were referred by their general practitioners for gait disorders, and by the outpatient memory consultation of the geriatric department to look for gait abnormalities.

Four main reasons for consultation (clinical subgroups) were identified: The subgroup of Recurrent Falls (RF) included patients with a history of 2 or more falls during the previous year. The Memory Impairment (MI) subgroup included patients with predominant memory complaints, without falls, or obvious gait impairment. The Gait Instability (GI) subgroup included patients who expressed a feeling of unstable gait, without recurrent falls, memory complaint or obvious gait disorders. The Cautious Gait (CG) subgroup included patients who moved slowly, with short strides, with little trunk movement, according to the description given by Snijders [29]. Enrolled patients should be able to walk unaided. Exclusion criteria included lack of French proficiency, less than 4 years of regular education, institutionalization, any acute medical condition requiring hospitalization within the past 6 months, and acute depression, defined by a Hospital Anxiety Depression (HAD) score of more than 11 out of 21 [30]. All patients were informed about the clinical procedure and provided informed consent prior to participation in the study.

### Outcome measures and baseline tests

The baseline assessment was carried out during the first consultation by the medical staff, and consisted of the following successive stages: (i) Self-questionnaires given to patients including the Dizziness Handicap Inventory and the Hospital Anxiety and Depression scale, (ii) balance and motor tests: One Leg Balance, Timed Up and Go, and Timed Chair Rise test (iii) Mini Mental State Examination (MMSE) for global cognition evaluation, (iv) complete geriatric examination with the identification of the different co-morbidities and medications, and (v) tests used for quantitative gait assessment as detailed below.

According to the results of the baseline assessment, patients would require the realization of a brain MRI examination and if necessary would be referred for a specialized consultation within the framework of the GIN. The identification of the main diagnoses and syndromes of gait disorders was carried out by the members of the GIN during their regular quarterly meetings.

Due to the diversity of diagnoses, homogeneous pathologies were grouped into 5 main pathological subgroups (except for fear of falling and Charcot Marie Tooth disease (peripheral neurological pathology)) as follows: osteoarthritis and myopathy, vestibular disease, mild cognitive impairment (MCI), CNS pathologies, and without diagnosis subgroups.

### Quantitative gait assessment

Gait performance under ST and DT task was assessed using a 3-D-acceleration sensor, a data logger and a computer program for processing the acceleration signal and calculating the gait parameters (Locometrix® [31, 32]). From a period of steady state walking of 21 s., the software calculated the stride frequency (Hz) and the stride regularity (dimensionless), which describes the similarity of vertical movements over successive strides. Further explanations on the calculation of the variables are described in previous papers [31, 32]. In addition, walking speed (m/s) was measured using a stopwatch. For the ST and DT, patients were asked to walk along a straight 30 m corridor, free of obstacles, using their regular walking shoes, at their preferred speed. First the ST trial consisted in walking at their usual pace. Secondly for the DT trial, patients walked at their usual pace whilst counting aloud backwards from 50 subtracting serial 1 s (one by one) [19, 20], with no instruction to prioritize the gait or cognitive task.

### Dual task cost

The Dual Task Cost (DTC) percentage figure was calculated for each gait variable as [((single task gait value—dual task gait value)/single task gait value)) x 100 [33–35]. The three new gait variables DTC (walking speed), DTC (stride frequency), and DTC (stride regularity) were used to search for differences between the different clinical subgroups and between the different main pathological subgroups.

### Quartile analysis

In the case of a high degree of dispersion or when pathological thresholds are not clearly defined, many works [36] have shown the relevance of categorizing the variables into quartiles (from minimum to first quartile, from first quartile to median, from median to third quartile and from third quartile to maximum). We applied the method of quartiles to the 3 new gait variables DTC (walking speed), DTC (stride frequency), and DTC (stride regularity). More specifically, we searched for the different motor phenotypes on the basis of the assumption that gait variables stride frequency and stride regularity can evolve independently in older patients (unpublished data). Consequently, we compared the respective evolution of DTC (stride frequency) and DTC (stride regularity). Three possibilities exist for this comparison: both variables will be in the same quartile, or each variable will be in a different quartile. Each of these 3 possibilities corresponds to one motor phenotype.

- N° 1 High value for DTC (stride frequency)—Low value for DTC (stride regularity) if the quartile of DTC (stride frequency) is higher than the quartile of DTC (stride regularity).
- N° 2 Same value for DTC (stride frequency) and DTC (stride regularity) if the quartiles are similar.
- N° 3 Low value for DTC (stride frequency)—High value for DTC (stride regularity) if the quartile of DTC (stride regularity) is higher than the quartile of DTC (stride frequency).

### Brain Imaging

Conventional brain imaging was carried out according to the recommendations used in the LADIS study [37] including the following sequences: T2-weighted images, including fluid-attenuated inversion recovery (FLAIR). Semi-quantitative score for white matter hyperintensities using the age-related white matter changes (ARWMC, scored 0–30, 0: no white matter hyperintensity; 30: maximum degree of white matter hyperintensities), [38], and Scheltens scored 0–4 (score 0: no atrophy, score 4: severe volume loss of hippocampus), [39] were identified.

### Data and statistical analysis

#### Sample size

When the total sample size across the 3 motor phenotype groups is 100, with unequal distribution across the groups (a minimum of 10 patients for the smallest group), a Kruskal-Wallis test will have 90% power to detect at a 1% level the difference in means characterized, by a variance of means of 20 assuming that the common standard deviation is 10. For the three identified motor phenotypes issued from quartile analysis, the values for DTC will be 5, 10, and 20 with a common standard deviation of 10. Consequently, the study will have the power to detect a difference of 5% across groups of Dual Task Cost for walking speed: DTC (walking speed), for stride frequency: DTC (stride frequency) and stride regularity: DTC (stride regularity).

All values are presented as a percentage for qualitative criteria and mean ± standard deviation (SD) for quantitative criteria. The differences in baseline gait parameters between main reasons for consultation (Gait Instability, Recurrent Falls, Memory Impairment and Cautious Gait) or other identified subgroups were compared with Chi square (C) or Fisher’s exact test (F) for qualitative criteria and Wilcoxon’s (W) or Kruskal-Wallis (KW) non-parametric tests for quantitative criteria (paired test for gait variable decrease).

As this study is mainly descriptive, no specific correction was used nor multiple comparisons. A *p*-value <1% is considered as highly statistically significant. The alpha value was set at *p* <0.05 to indicate statistical significance, unless otherwise noted. Software SAS® version 9.4 was used to perform all statistical analyses.

## Results

### Main reason for consultation

One hundred and three patients (mean age 76.3 ± 7.2 years); female 58 (56%), were included consecutively in the study. As expected, males were significantly different from females in height and Body Mass Index (BMI) (W: 0.01) but no differences were observed in age (W: 0.16), MMSE (W: 0.26), or number of medications (W: 0.54). The following four clinical subgroups were identified according to the main reason for consultation:

- Gait Instability (GI) (*n* = 46 (45%)), (F = 22, M = 24),
- Recurrent falls (RF) (*n* = 30 (29%)), (F = 23, M = 7),
- Memory impairment (MI) (*n* = 19 (18%)) (F = 7, M = 12),
- Cautious gait (CG) (*n* = 8 (8%)), (F = 6, M = 2).

Demographic and clinical characteristics are presented in Table 1. No differences in age (KW: 0.23) or number of medications (KW: 0.14) were found between clinical subgroups. Significant differences in sex (C: 0.02), height (KW: 0.01), BMI (W: 0.05), and MMSE (KW: 0.01) were observed, especially for the group of Cautious Gait that exhibited the lowest MMSE scores.

**Table 1:** Males were different from females for height and BMI (W: 0.01), but not for age (W: 0.16), MMSE (W: 0.26), or number of medications (W: 0.54)

	Overall	Gait Instability			Recurrent Falls			Memory Impairment			Cautious Gait		
Mean ± SD Median	(*n* = 103)	F (*n* = 22)	M (*n* = 24)	Overall (*n* = 46)	F (*n* = 23)	M (*n* = 7)	Overall (*n* =30)	F (*n* = 7)	M (*n* = 12)	Overall (*n* = 19)	F (*n* = 6)	M (*n* = 2)	Overall (*n* = 8)
Age (Years)	76 ± 7 77	77 ± 8 78	76 ± 5 74	76 ± 8 77	77 ± 8 78	80 ± 4 79	77 ± 7 78	76 ± 5 78	75 ± 6 76	75 ± 5 76	81 ± 5 81	73 ± 16 73	79 ± 9 81
Height (cm)	162 ± 10 161	157 ± 5 158	173 ± 7 173	166 ± 10 165	155 ± 70 156	163 ± 4 164	157 ± 7 157	158 ± 5 156	170 ± 6 170	166 ± 8 167	155 ± 4 156	164 ± 16 164	157 ± 8 156
BMI (kg/m2)	25 ± 4 24	25 ± 5 25	27 ± 4 26	26 ± 5 26	24 ± 4 23	26 ± 3 26	24 ± 3 24	22 ± 3 21	25 ± 3 24	24 ± 3 24	25 ± 4 26	31 ± 3 31	27 ± 4 28
MMSE	27 ± 3 27	28 ± 1 28	27 ± 4 28	28 ± 3 28	26 ± 3 28	25 ± 3 26	26 ± 3 27	27 ± 1 27	26 ± 3 27	26 ± 2 27	24 ± 5 24	22 ± 6 22	23 ± 5 24
Number of medications	5 ± 3 5	5 ± 3 4	5 ± 3 5	5 ± 3 5	5 ± 2 5	5 ± 3 4	5 ± 2 5	3 ± 3 1	4 ± 4 4	4 ± 3 3	7 ± 3 6	5 ± 1 5	6 ± 3 6

### Dizziness handicap inventory, timed up and go, one leg balance test, and timed chair rise test

The Dizziness Handicap Inventory is an auto questionnaire that helps the diagnosis of vestibular origin. Results of the TUG, according to clinical subgroup were as follows: MI: 16 ± 3 s, GI: 21 ± 6 s, RF: 23 ± 7 s, CG: 34 ± 15 s, (KW < 0.01). One leg balance and timed chair rise test were useful measurements to tailor rehabilitation for balance, and limb strength, respectively.

### Main diagnoses, syndromes and main pathological subgroups

Table 2 shows a wide variety of established diagnoses for included patients, and the main pathological subgroups. Neurological disorders were largely predominant (84/103) followed by musculoskeletal disorders (11/103), mainly osteoarthritis. Six patients were diagnosed with a vestibular disorder, 3 with Parkinson’s Disease, and only 1 patient with a predominant symptom of fear of falling. Finally, 8 patients were undiagnosed. Diagnoses differed significantly according to sex (F: 0.03) but not according to clinical subgroups (C: 0.57). According to the established main diagnoses of gait disorders in included patients, the following main pathological subgroups were identified: osteoarthritis and myopathy (*n* = 11), vestibular diseases (*n* = 6), MCI (*n* = 24), CNS pathologies, (*n* = 51), and without diagnosis (*n* = 8).

**Table 2:**
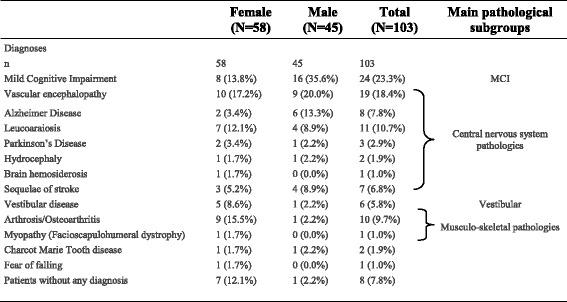
Diagnoses differed according to sex (F: 0.03), but not according to clinical subgroups (C: 0.57)

### Gait variables, under Single and Dual Task, (mean value and SD) according to main clinical subgroups (Table 3)

**Table 3:** All gait variables under DT had significantly lower values than under ST (KW < 0.0001). Walking speed and Stride Regularity decreased similarly between clinical subgroups, and allowed the grading of biomechanical severity of subgroups from the least to the most serious as follows: memory impairment, gait instability, recurrent falls, cautious gait

N	Memory Impairment (*n* = 19)		Gait Instability (*n* = 46)		Recurrent Falls (*n* =30)		Cautious Gait (*n* = 8)		*p*-value (Kruskal-Wallis)	
Mean ± SD Median	Single Task	Dual Task	Single Task	Dual Task	Single Task	Dual Task	Single Task	Dual Task	Single Task	Dual Task
Walking Speed (m/s)	1.2 ± 0.2 1.2	1.0 ± 0.3 1.0	1.0 ± 0.2 1.0	0.9 ± 0.2 0.9	0.9 ± 0.2 0.9	0.8 ± 0.3 0.7	0.7 ± 0.2 0.7	0.6 ± 0.3 0.6	<0.01	<0.01
Stride Frequency (Hz)	0.92 ± 0.07 0.92	0.80 ± 0.14 0.81	0.92 ± 0.09 0.9	0.82 ± 0.09 0.84	0.88 ± 0.09 0.87	0.78 ± 0.12 0.78	0.90 ± 0.08 0.90	0.80 ± 0.11 0.83	0.04*	0.33
Stride Regularity (dimensionless)	258 ± 54 269	195 ± 54 205	214 ± 47 217	170 ± 51 165	199 ± 56 210	159 ± 62 165	147 ± 55 145	115 ± 73 107	<0.01	0.05

For stride frequency and single task, non-parametric KW test is significant (*p* = 0.04) because mean scores (rank) is lower for recurrent falls and cautious gait (mean rank = 41) than memory impairment and gait instability (mean rank = 58)

Male and female patients showed a significant difference for walking speed (W: 0.02) during ST but not for stride frequency (W: 0.40) or stride regularity (W: 0.25). No significant differences were observed between the sexes for the 3 gait variables during DT: walking speed (W: 0.11), stride frequency (W: 0.10), and stride regularity (W: 0.66). All gait variables (walking speed, stride frequency, and stride regularity) under DT had significantly lower values than under ST conditions (KW < 0.0001). Walking speed was different between clinical subgroups under both ST and DT (KW < 0.01for both conditions). Stride regularity was also different between clinical subgroups under both ST and DT (KW < 0.01 and 0.05, respectively). These two variables decreased similarly between clinical subgroups, which allowed the biomechanical severity of subgroups to be classified from least to most serious as follows: MI, GI, RF, and CG. SF showed different median values between subgroups only under ST (KW 0.04). Furthermore, a paradoxical improvement in gait variables under DT was observed in some patients: walking speed in 1 patient, stride frequency in another 1 patient, and stride regularity in 15 patients out of 103 (mean −8.5 ± −6.2). We considered that the paradoxical improvement is due to the cueing effect.

### Dual Task Cost for each gait variable according to clinical subgroups (Table 4)

**Table 4:** In a multivariate analysis of a variance model we observed a strong gait variable effect (*p* < 0.001) and no clinical subgroup effect (*p* = 0.58)

Mean ± SD Median	Memory Impairment (*n* = 19)	Gait Instability (*n* = 46)	Recurrent Falls (*n* =30)	Cautious Gait (*n* = 8)	Total (*n* = 103)
DTC Walking Speed (%)	14.6 ± 14.1 9.8	13.5 ± 9.5 12.0	16.3 ± 13.6 12.2	15.5 ± 14.3 11.8	14.7 ± 12.0 12.1
DTC Stride Frequency (%)	13.5 ± 12.3 10.5	10.2 ± 7.9 8.2	10.7 ± 8.9 9.1	11.4 ± 15.5 7.2	11.1 ± 9.7 9.2
DTC Gait Regularity (%)	24.8 ± 24.9 24.2	19.6 ± 19.9 18.2	20.9 ± 21.8 16.8	22.4 ± 29.7 17.9	21.1 ± 22.0 18.4

Dual task cost for each gait variable did not differ between the sexes: DTC (walking speed) (W 0.39), DTC (stride frequency) (W 0.45), DTC (stride regularity) (W 0.29), or clinical subgroups: DTC (walking speed) (KW 0.90), DTC (stride frequency) (KW 0.70), DTC (stride regularity) (KW 0.88). The mean values of DTC were different for each gait variable (multivariate analysis of variance model *p* < 0.001): DTC (stride frequency) = 11.1 ± 9.7%, DTC (walking speed) = 14.7 ± 12.0%, DTC (stride regularity) = 21.1 ± 22.0%. A strong correlation (*p* < 0.001) was observed between the mean values of dual task cost for each gait variable: R2 DTC (walking speed)/ DTC (stride frequency) = 0.74, R2 DTC (walking speed)/DTC (stride regularity) = 0.69, R2 DTC (stride frequency)/ DTC (stride regularity) = O.69.

### Dual task cost for each gait variable according to main pathological subgroups (Table 5)

**Table 5:**
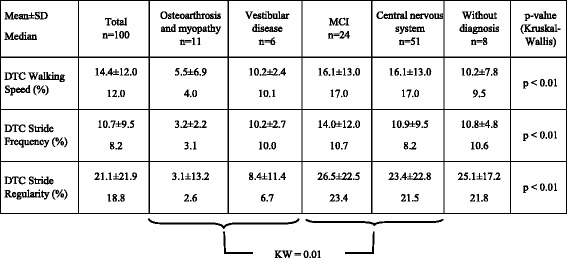
Dual Task Cost for each gait variable differed between the main pathological subgroups (KW <0.01). The comparison between the 2 subgroups with low DTC (musculoskeletal diseases, vestibular diseases) combined on one side and the 2 groups (MCI, CNS pathologies) with high DTC values on the other showed significant differences in DTC for each gait variable (KW: 0.01)

Dual task cost for each of the three gait variables was different between the main pathological subgroups (KW < 0.01). The lowest values of DTC were observed in the subgroup of musculoskeletal diseases whilst the highest values were observed in the MCI subgroup as well in CNS pathology subgroups. When the 2 subgroups with low DTC values (musculoskeletal diseases, vestibular diseases) were combined on one side and the 2 subgroups (MCI, CNS pathologies) with high DTC values on the other, the comparisons of DTC for walking speed, stride frequency, and stride regularity showed significant differences at the 1% threshold.

### Quartile analysis and Identification of motor phenotypes Fig. 1

**Fig. 1:**
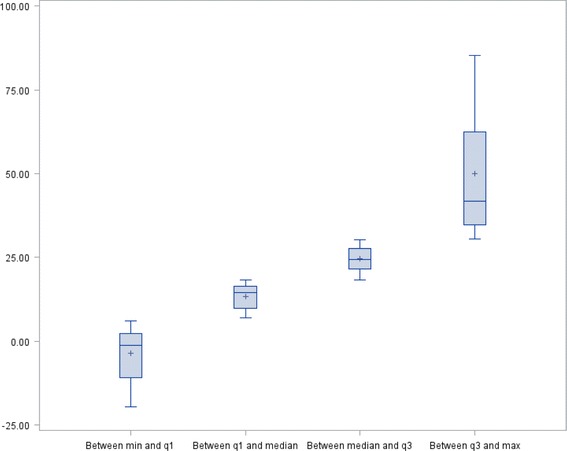
The quartile analysis illustrates the great dispersion of the values of the Dual Task Cost for Stride Regularity

**Fig. 2:**
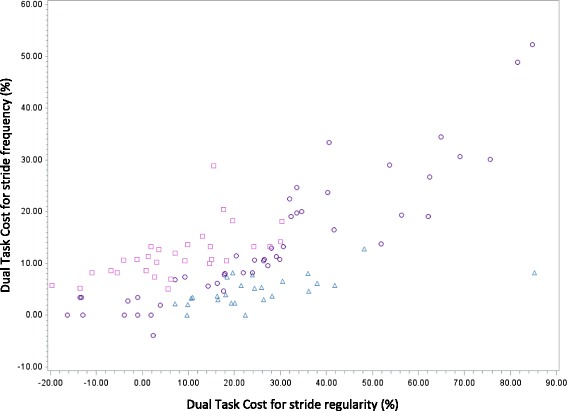
Motor phenotypes identified on the basis of quartile analysis of Dual Task Cost for Stride Frequency and Stride Regularity (KW <0.01, *r* = 0.69, *p* < 0.0001).  High value of DTC for Stride Frequency—Low value of DTC for Stride Regularity (*n* = 30). N°2:  Same value of DTC for Stride Frequency and Regularity (*n* = 47). N°3:  Low value of DTC for Stride Frequency—High value of DTC for Stride Regularity (*n* = 26)

The quartile analysis illustrates the dispersion of the values of DTC for each gait variable (Fig. 2 (Box Plot of DTC for stride regularity)). The box plots of DTC for walking speed and stride frequency are similar to those of DTC for stride regularity. Moreover, Additional file 1: Table S1 shows the mean values of different quartiles for each variable; DTC (walking speed), DTC (stride frequency), and DTC (stride regularity). For each of these 3 variables, the quartiles are different. However, for each quartile the values are different between the three variables (the three variables have different values). This method of analysis shows that it is possible to quantify the DTC value for each of these variables and demonstrated that the values differ according to the considered variable. The quartile analysis of DTC for stride frequency and stride regularity allowed the identification of three different motor phenotypes (KW < 0.01, *r* =0.69, *p* < 0.0001). The first motor phenotype corresponded to patients in a quartile of DTC for stride frequency higher than that of DTC for stride regularity (ex q2 and q 1, respectively, *n* = 30), the second comprised patients in similar quartiles of DTC for both gait variables (*n* = 47); and the third motor phenotype included patients in a quartile of DTC for stride frequency lower than that of DTC for stride regularity (ex q1 and q2, respectively, *n* = 26). The three motor phenotypes did not show differences for MMSE or established diagnoses. Patients of the third phenotype were older (*p* = 0.02) with an increased number of medications (*p* = 0.04).

### Brain MRI

Only 77 patients out of 103 underwent brain MRI due to the presence of absolute contra-indications such as pacemaker or claustrophobia, or patient refusal. Results showed an Age Related White Matter Changes score (ARWMC) of 3.5 ± (3.9), range (0-18/30); and a Scheltens total score (right and left) of 3.2 ± 1.8 (range 0-8/8). There was no difference between the male and female patients (KW: 0.92 and 0.09, respectively). No differences between the 4 clinical subgroups were noted for ARWMC and Scheltens scores (KW: 0.42 and 0.59, respectively). No differences were noted between the 3 motor phenotypes for ARWMC, Scheltens scores increased steadily across the three motor phenotypes: first (2.6 ± 1.6); second (3.3 ± 1.6) third phenotype (4.0 ± 1.9); (*p* = 0.05).

## Discussion

### Gait disorders in the Elderly

Our study included 103 elderly patients referred by their general practitioners or by the outpatient memory consultation of the geriatric department to look for gait disorders. Patients complaining of gait instability expressed a feeling of unstable gait, without falls, memory impairment or obvious gait disorder were the main clinical subgroup. Therefore, clinicians should identify gait instability as a major clinical symptom, and a multi-disciplinary approach will be necessary to manage this complaint as it is associated with a large number of diseases.

### Diagnosis

Abnormal gait resulting from neurological conditions was largely predominant as shown by other studies [40]. The differences according to sex were mainly due to the higher prevalence of osteoarthritis in female patients, which is consistent with the literature [41]. White matter lesions were found in some of the patients having gait disorders, in the absence of underlying neurological pathology [42]. Parkinson’s disease and dizziness were responsible for gait disorders in only 3 and 6 patients, respectively, despite the high frequency of these two conditions in gait disorders. This can be explained by the fact that patients with Parkinson’s disease are usually referred to neurologists, and those suffering from dizziness are referred to ENT specialists. Surprisingly, the diagnosis of fear of falling, which is a prime concern of many elderly patients with unstable gait [43] was identified as the main cause of gait disorders in only one patient. Nevertheless, fear of falling may be present in a larger number of patients but may remain hidden by another cause of gait disorders such as MCI. Finally, no etiology was identified for gait disorders in 8 patients. The main causes of gait disorders were similar through the four clinical subgroups (C = 0.85). This finding highlights the importance of cognitive and motor interactions in elderly subjects, the relevance of gait analysis under single and dual-task conditions in the assessment of gait disorders in elderly people, and the great clinical value of gait instability as a symptom.

### Gait Assessment and gait variables

#### Gait assessment

Wireless reliable triaxial accelerometers are widely used for gait analysis in ambulatory conditions [20–22, 44, 45]. From raw acceleration data recorded next to the body mass center, gait characteristics, such as stride frequency and stride regularity may be calculated. Reliability tests such as precision, accuracy [46], effective test-retest reliability and reproducibility [32, 47, 48] during stabilized walking were carried out. Accelerometers were used as they are well adapted to continuous measurement over a period of stabilized walking, which must consist of at least 20 gait cycles in frail elderly people, especially if gait variability has to be assessed [49].

#### Walking speed

Walking speed remains the easiest gait variable to measure [50]. In clinical settings, walking speed is a meaningful validated marker of functional status and health in older adults [5]. A walking speed threshold of 1 m/s was the established threshold of adverse health outcomes in older adults [51]. In elderly people, a threshold greater than 1 m/s would be considered normal. In our study, walking speed under ST was ≥ 1 m/s in both GI and MI subgroups, which demonstrates that measuring walking speed is insufficient for the MI and CI clinical subgroups, and that other gait variables such as stride frequency and stride regularity have to be measured. Nevertheless, walking speed remains an interesting variable that reflects the severity of symptoms and their clinical impact, allowing us to grade the four subgroups by order of severity as follows: MI, GI, RF, and CG.

#### Stride Frequency

The rhythmic movement pattern of human gait is established at the level of the spinal cord where the so-called central pattern generators are under the control of the supraspinal network. [52]. Stride frequency is often integrated as a rhythmic factor, in order to reduce numerous gait variables to three main factors (pace, rhythm, and variability factors [6]). Few studies have examined the clinical importance of a decreased stride frequency, by itself, in clinical practice. A reduced stride frequency has been found in patients with subcortical vascular encephalopathy [53]. In the current study, there was no difference between the four clinical subgroups in terms of stride frequency mean values under ST or DT. This is in contrast with the finding of a progressive decrease in walking speed and stride regularity. This point raises two questions: the independency of stride frequency with respect to walking speed [54] and stride regularity, and the clinical significance of a reduced stride frequency.

#### Gait Regularity or Gait variability

Intra-individual gait variability, which measures the shortest fluctuation in gait over a period of seconds to minutes within a single session, has been described as a consistent clinical marker for high risk elderly patients: increased risk of falling, developing mobility disability, or dementia [55]. Gait variability can be measured by means of spatio-temporal measurements such as step length, width, step time and stance time, stride time and swing time. They are markers of impaired control, arhythmicity and unsteadiness [56]. Gait variability can also be measured from trunk acceleration, and provides information related to balance control during walking [57]. Gait variability measured by autocorrelation functions from trunk accelerations is highly reliable [58]. Gait variability is measured by means of a coefficient of variation according to the gait variable selected (CV = SD/menx100). Gait variability increases when there is a decrement of gait. The variability of gait measured by the Locometrix® system (autocorrelation functions) is named regularity. Gait regularity decreases when there is a decrement of gait. For healthy elderly people aged over 70, there is no difference between the sexes and the mean value used in clinical practice is 300 ± 30 [32].

Compared to standards established within a control population (300 ± 30), the gait regularity is significantly decreased for the 4 clinical subgroups under ST conditions including the 2 clinical subgroups MI and GI in which walking speed is normal (>1 ms). This is in accordance with the fact that gait variability is the most prominent gait abnormality with respect to velocity [1], and is more sensitive in predicting falling when compared to gait speed [59]. Moreover, it is important to note that patients referred for memory disorders had a loss of stride regularity under both ST and DT, despite a walking speed > 1 m/s. This finding raises the question of adding gait analysis under both ST and DT to memory consultation for assessing the cognitive resource that regulates gait [60]. Whatever the considered situation ST or DT, there is a progressive reduction of the gait regularity between the 4 clinical subgroups in the following order: MI, GI, RF and CG. This grading is the same as that for walking speed.

The paradoxical gait regularity improvement (15/103) observed under dual-task conditions leads us to suggest the possibility of the cueing effect. The cueing effect refers to the impact of a rhythmic auditory stimulation to improve walking parameters. This has already been proven in many pathological situations such as Stroke and Parkinson’s disease [61, 62]. In our study, the observation of improving regularity under the dual task of spoken countdown raises the question of the mechanism of the observed cueing effect. This could be of internal origin by the attention paid to the countdown, or it may be of external origin by hearing the countdown. Complementary studies are necessary to confirm these results and to better understand them.

#### Dual Task Cost and quartile analysis

Walking deterioration resulted in a reduction of the 3 variables (walking speed, stride regularity and stride frequency). The used formula ((single task gait value—dual task gait value)/single task gait value)) x 100 showed a positive value, which is consistent with the cost concept of DTC. There is a lack of consensus concerning the arithmetic attentional demanding task. Various serial subtractions have been proposed: S-1[19, 20, 35], S- 3, or S- 7 [18, 33, 34, 63]. Despite the fact that arithmetic (attentional) task with a high cognitive demand (S-7) has been reported with the highest sensitivity [26, 64], we selected the simplest arithmetic one: i.e. S-1, because it has previously been validated in elderly patients with cognitive disorders [19, 20, 26, 35]. Another reason is that in patients with dementia who have a broad range of pathologies, a simple cognitive task is needed for a reliable dual-task assessment [64]. Moreover, the walking test used in the current study consists in walking over a distance of 30 m requiring a sustained attention that is known to be an additional stress to the dual task [65, 66].

Anxiety and depression which have a negative impact on cognitive and motor performances [25, 33], had, in the current study, limited effect on patients’ performances under DTC, since the mean score of HAD was less than 11.

DTC in each gait variable did not vary in the 4 clinical subgroups (Table 4), whilst the mean DTC showed a gradual significant increase by going from stride frequency to stride regularity through walking speed. Accordingly, the identification of a critical threshold for DTC should consider the variability of DTC values in the different gait variables. To date, cut-off for DTC (walking speed), DTC (stride frequency), and DTC (stride regularity), which allows on the one hand the differentiation of healthy populations from pathological ones, and on the other hand the identification of their clinical significance is not known [1]. Nevertheless, quartile analysis may represent an approach for this goal. Furthermore, it is of interest to note that, in daily clinical practice, DTC allows the differentiation between gait disorders due to peripheral pathologies and those secondary to central neurological pathologies, whatever the variable: DTC (walking speed), DTC (stride frequency), or DTC (stride regularity) (Table 5).

DTC are similarly high for each gait variable between the main pathological groups (MCI and CNS pathologies) due to impairment of cognitive functions in these 2 subgroups, which results in marked walking decline. Meanwhile, it is not possible to differentiate DTC according to pathologies (MCI, vascular encephalopathy, Alzheimer’s disease) because of the heterogeneity and the degree of severity of the corresponding pathologies [20, 34, 67]. Nevertheless, DTC for the different gait variables are significantly lower for sequelae of stroke (DTC (walking speed): 9.9 ± 7.8, DTC (stride frequency): 6.2 ± 8.1, DTC (stride regularity) 8.4 ± 17.2, in accordance with the fact that the prevalence rate of cognitive impairment in patients with a stroke history varies from 24 to 33% [68]. Moreover, a low DTC for osteoarthrosis, myopathy, and vestibular disease is in line with the absence of cognitive impairment in gait disorders linked to these pathologies.

#### Motor phenotypes and Brain MRI

The identification of motor phenotypes raised logical questions, especially concerning the clinical value of these motor phenotypes, and the presence of a link between these ones and brain imaging findings. Such correlations have already been shown by some studies [26] in order to identify motor phenotypes in patients with MCI. On the other hand, other studies have found a link between increased gait variability and a reduced hippocampal volume [28]. In our study, no correlation was found between these motor phenotypes and clinical subgroups, nor main pathological subgroups, nor white matter hyperinsensity. We noted a reduced hippocampal volume from the first to the third motor phenotype which was characterized by a quartile stride regularity higher than that of stride frequency, this finding has to be confirmed by other studies. Therefore, conventional brain MRI is insufficient for the evaluation of the clinical impact of White Matter hyperintensity since clinical manifestations depend more on White Matter hyperintensity localizations rather than their volume [69]. Moreover, according to Allali, links between motor phenotypes and brain imaging necessitate different neuroimaging techniques, including structural and functional magnetic resonance imaging as well as functional near-infrared spectroscopy [70].

#### Limitations and future developments

It is preferable to increase the number of patients in each clinical subgroup to reach at least 30 patients per subgroup. Cautious gait has to be discussed according to the characteristics of the so-called High-Level Gait Disorder [71]. A more detailed cognitive evaluation using additional assessment tools other than MMSE alone should be carried out in order to screen the cognitive status of patients in the primary outpatient consultation [72], for instance the Montreal Cognitive Assessment, which takes into account the evaluation of executive functions.

Another important issue to address is the best gait variables to use in assessing gait disorders. Walking speed is an overall measure that is of great importance due to its simplicity and high clinical relevance. However, it is the resultant of stride length and stride frequency, which are controlled by a neurological center other than that controlling walking speed. Stride length is assumed to be controlled supraspinally by a basic output from basal ganglia to the supplementary motor area [73]. Therefore, future studies must take into account both stride frequency and stride length rather than walking speed alone.

The nature of dual tasking influences the DT decrements [33], as well as the prioritization, which remains a great difficulty in clinical practice. The factors that contribute to the dual tasking changes in performance such as education level, physical activity, depression status [74] could be more detailed. The present findings strengthen the evidence that gait analysis in older people must be carried out using the dual task paradigm in clinical routines [21, 60], with an additional point which will be to measure the concurrent task performance [75, 76].

The strength of this study is the novel clinical approach and the validated gait test procedures that were used. To our knowledge, this is the first time that subgrouping of patients has been carried out according to their motor characteristics, using a DT for gait analysis, and taking into account DTC (stride frequency) and DTC (stride regularity). It will be necessary for these results to be replicated, and then their clinical values to be evaluated by means of case-control follow-up studies. Moreover, the identification of the links between brain imaging and motor phenotypes, according to different grades of the DTC decrement in stride frequency and stride regularity, necessitates more powerful brain imaging than the conventional one. Finally, as a result of reduced cognitive performance in the different clinical subgroups in the current study, it is of interest to combine cognitive therapy with motor intervention in clinical practice to enable older patients to move with greater security and reduce fall risk [72].

## Conclusions

In older adults, gait instability without falling is a frequent complaint, and has to be identified and taken into account by the clinician. This complaint, as well as recurrent falls, memory impairment, and cautious gait necessitates a multi-disciplinary approach. Data taken from scientific literature revealed that gait analysis under single and dual-task conditions allows the measurement of the DTC, which is related to the cognitive resource of the patient. The measure of DTC for each gait variable can differentiate between peripheral pathologies and CNS pathologies in the elderly. Furthermore, DTC was found to be specific for each variable, it increases significantly from stride frequency to stride regularity passing by walking speed. Cut-off values of dual task cost were identified by quartile analysis. Three motor phenotypes were identified by means of DTC for stride frequency and stride regularity. One of these 3 phenotypes, included patients in a quartile of DTC for stride frequency lower than that for DTC for stride regularity, was characterized by a higher MRI Scheltens score. Future prospective case-control studies, with extensive follow up, taking into account DTC for stride frequency and stride regularity should be carried out to replicate these results and to identify a meaningful cut-off for clinical applications. Finally, one must look for more correlations between motor phenotypes and brain MRI abnormalities.

## Additional file

Additional file 1:
**Table S1.** Dual Task Cost (DTC) according to Quartile analysis of DTC for walking speed, DTC for stride frequency and DTC for stride regularity. (DOCX 13 kb)

## Abbreviations

ARWMC: Age related white matter change
BMI: Body Mass Index
C: Chi square test
CG: Cautious gait
CNS: Central nervous system
DT: Dual task
DTC: Dual task cost
DTC (stride frequency): Dual task cost for stride frequency
DTC (stride regularity): Dual task cost for stride regularity
DTC (walking speed): Dual task cost for walking speed
F: Fisher exact test
GI: Gait instability
GIN: Gait instability network
HAD: Hospital Anxiety Depression
KW: Kruskal-Wallis test
MCI: Mild cognitive impairment
MI: Memory impairment
MMSE: Mini Mental State Examination
RF: Recurrent falls
ST: Single task
W: Wilcoxon test
