# Supplementary material for: Gait disorders in the elderly and dual task gait analysis: a new approach for identifying motor phenotypes
Source: J Neuroeng Rehabil. 2017 Jan 31;14:7. doi: 10.1186/s12984-017-0218-1 (PMC5282774; doi:10.1186/s12984-017-0218-1)
Supplement: Additional file 1: — Table S1. Dual Task Cost (DTC) according to Quartile analysis of DTC for walking speed, DTC for stride frequency and DTC for stride regularity. (DOCX 13 kb) [file 12984_2017_218_MOESM1_ESM.docx]

| **Table S1**  **Dual Task Cost (DTC) according to Quartile analysis of DTC for Walking Speed (DTC WS), Stride Frequency (DTC SF), and Stride Regularity (DTC SR)** | | | | | | | | | | | |
| --- | --- | --- | --- | --- | --- | --- | --- | --- | --- | --- | --- |
| DTC Walking Speed | Minimum | Maximum | Mean±SD | DTC Stride Frequency | Minimum | Maximum | Mean±SD | DTC Stride Regularity | Minimum | Maximum | Mean±SD |
| First quartile (Q1)  N=25 | -3.1 | 6.6 | 2.2±2.7 | First quartile (Q1)  N=24 | -3.9 | 4.0 | 1.8±1.9 | First quartile (Q1)  N=26 | -19.7 | 6.1 | -3.6±7.5 |
| Second quartile (Q2)  N=25 | 6.9 | 12.0 | 9.5±1.7 | Second quartile (Q2)  N=24 | 4.7 | 8.2 | 6.5±1.2 | Second quartile (Q2)  N=25 | 7.0 | 18.2 | 13.4±3.9 |
| Third quartile (Q3)  N=27 | 12.1 | 20.2 | 15.5±2.9 | Third quartile (Q3)  N=26 | 8.2 | 13.2 | 10.5±1.5 | Third quartile (Q3)  N=26 | 18.4 | 30.3 | 24.6±3.7 |
| Fourth quartile (Q4)  N=26 | 20.3 | 54.3 | 30.7±10.7 | Fourth quartile (Q4)  N=29 | 13.3 | 52.3 | 22.9±10.0 | Fourth quartile (Q4)  N=26 | 30.5 | 85.3 | 49.9±18.0 |

The quartile of DTC for each gait variable (WS, SF,SR) is different. For each quartile the DTC values are different between each gait variable.
